# MetaCYP: a unified framework for prediction of cytochrome P450 metabolic sites and reaction types via multimodal deep learning

**DOI:** 10.3389/fchem.2026.1869559

**Published:** 2026-08-12

**Authors:** Jiamin Chang, Xuhai Huang, Boxue Tian

**Affiliations:** MOE Key Laboratory of Bioinformatics, State Key Laboratory of Molecular Oncology, Beijing Frontier Research Center for Biological Structure, School of Pharmaceutical Sciences, Tsinghua University, Beijing, China

**Keywords:** cytochrome P450, deep learning, metabolic site, prediction, reaction type

## Abstract

**Introduction:**

Cytochrome P450 (CYP) enzymes are the predominant drug-metabolizing proteins in humans, collectively governing the structural transformation of drugs and xenobiotics while directly shaping their pharmacological activity and toxicological profiles. Accurate prediction of CYP–substrate reaction sites and reaction types is therefore central to drug discovery and metabolic risk assessment. Existing computational models, however, largely depend on intrinsic molecular properties or hardcoded reaction rules, constraining their generalization across CYP isoforms.

**Methods:**

Here we present MetaCYP, a multimodal deep learning framework that predicts bonds of metabolism (BoMs) and reaction types in CYP-mediated biotransformation. MetaCYP encodes CYP amino acid sequences with the protein language model ESM-2 and extracts bond-level substrate features using Uni-Mol, integrating both modalities through an attention-based cross-modal fusion mechanism that captures enzyme–substrate interactions.

**Results:**

This architecture enables a single unified model to resolve isoform-specific catalytic selectivity for identical substrates. MetaCYP achieves state-of-the-art performance in BoM prediction (MCC: 0.741; ROC-AUC: 0.956) and reaction type prediction (MCC: 0.796; ROC-AUC: 0.946), outperforming current benchmarks.

**Discussion:**

MetaCYP establishes a unified deep learning framework that models reaction sites and reaction types from enzyme–substrate information, offering a mechanistically grounded and interpretable tool for elucidating CYP catalytic selectivity. Its capacity to improve the accuracy of ADME property predictions, alongside its scalability across isoforms, positions it as a practical resource for early-stage drug screening, metabolic risk assessment, and rational drug design.

## 1 Introduction

Cytochrome P450 (CYP) enzymes are a superfamily of heme-containing monooxygenases and responsible for over 90% of the oxidative metabolism of drugs and xenobiotics in humans (Esteves et al., 2021; Hirao et al., 2025; Lee et al., 2024). Their interactions with substrate molecules dictate both the structural identity of metabolic products and downstream biological consequences, including alterations to drug efficacy and half-life, drug–drug interactions (DDIs), and adverse toxicological effects (Hakkola et al., 2020; Rendic and Guengerich, 2024; Xie et al., 2016). The nature of these metabolites is therefore a critical consideration throughout drug discovery, clinical pharmacotherapy, and toxicological risk assessment. Reliable prediction of sites of metabolism (SoMs), reaction types, and product structures would substantially accelerate drug development and reduce experimental burden (de Bruyn Kops et al., 2019; Zaretzki et al., 2013).

Computational strategies for modeling CYP–molecule interactions have progressively displaced labor-intensive experimental assays, enabling high-throughput screening and mechanistic interrogation (Chen et al., 2024; Fu et al., 2024; Hunt et al., 2018; Plonka et al., 2021; Tian et al., 2018; Tyzack and Kirchmair, 2019; Xiao and Hirao, 2025). DR-Predictor and IDSite leverage molecular docking to derive geometric and energetic features of protein–ligand complexes, subsequently applying multiple-instance learning or parameter fitting to infer SoMs (Huang et al., 2013; Li et al., 2011). SMARTCyp employs density functional theory (DFT)-derived bond dissociation energies alongside accessibility descriptors within a fragment-based scoring framework (Olsen et al., 2019; Rydberg et al., 2010). Classical machine learning models, including FAME, RS-WebPredictor, and ADMET Predictor (Simulations Plus, Inc, 2020), encode molecular descriptors such as charge distribution, topological indices, local atomic environments, and molecular fingerprints, and apply random forests, support vector machines, or gradient boosting to estimate SoM probabilities (Sicho et al., 2019; Zaretzki et al., 2013). More recently, GLMCyp advanced the field by integrating 2D/3D molecular representations with protein features through graph attention networks for bond-level BoM prediction (Huang et al., 2025). End-to-end pipelines such as CyProduct and DeepMetab extend this paradigm further by coupling substrate prediction, SoM identification, and metabolite generation within a single framework, typically combining graph neural networks (GNNs) with rule-based chemical constraints (Tian et al., 2021; Zhou et al., 2025). BioTransformer and ADMET Predictor similarly rely on SMIRKS reaction templates to define transformations and infer reaction types via rule matching (Simulations Plus, Inc, 2020; Wishart et al., 2022). MetaReact treats CYP-mediated metabolism as a direct product generation task, encoding enzyme identity as a categorical variable without explicit protein sequence modeling and without predicting BoMs or reaction types (Wang et al., 2026).

Despite their merits, these approaches share fundamental limitations. Physics-based methods, molecular docking, pharmacophore modeling, and quantum chemical calculations, provide mechanistic interpretability but are computationally prohibitive at scale (Li et al., 2011; Rydberg et al., 2010; Zaretzki et al., 2013). Most machine learning and deep learning models are task-specific, targeting either substrate binding or SoM prediction in isolation, and rely on heavily engineered molecular features that incompletely capture enzyme–substrate interactions (Sicho et al., 2019; Tian et al., 2021). Although GLMCyp incorporates protein information, it remains limited to reaction-site prediction and does not model reaction types or metabolite generation (Huang et al., 2025). End-to-end models, conversely, depend on predefined reaction rules to assign reaction types, limiting their coverage and adaptability to novel scaffolds or rare metabolic pathways (Zhou et al., 2025). Critically, rule-based systems cannot encode enzyme-specific selectivity: the same BoM may undergo hydroxylation catalyzed by one CYP isoform yet dealkylation by another, a distinction that static rules are structurally unable to resolve. Because CYP-mediated metabolism emerges from the complex interplay between enzyme and substrate, methods grounded solely in molecular reactivity or fixed transformation rules are inherently insufficient for accurate reaction type and metabolic outcome prediction.

To address these limitations, we introduce MetaCYP, a multimodal deep learning framework for predicting CYP-mediated reaction sites and reaction types. MetaCYP accepts CYP amino acid sequences and substrate SMILES as dual inputs, encoding them with ESM-2 and Uni-Mol (Lin et al., 2023; Zhou et al., 2023), respectively, to obtain high-dimensional protein and bond-level molecular representations. An attention-based cross-modal fusion module integrates these representations to model enzyme–substrate interactions explicitly (Vaswani et al., 2017). A multi-layer perceptron (MLP) head then predicts BoMs and reaction types from the fused representation. By incorporating both CYP sequence and substrate SMILES, MetaCYP captures isoform-dependent reaction selectivity and generalizes across multiple CYP isoforms within a single model, without reliance on reaction rules or manually engineered features. This framework addresses the core challenge of predicting metabolic outcomes in complex, enzyme-specific contexts and provides a scalable foundation for CYP-mediated drug metabolism prediction, with direct applicability to early-stage drug screening, ADME evaluation, rational drug design, and metabolic risk assessment.

## 2 Methods

### 2.1 MetaCYP framework

**FIGURE 1:**
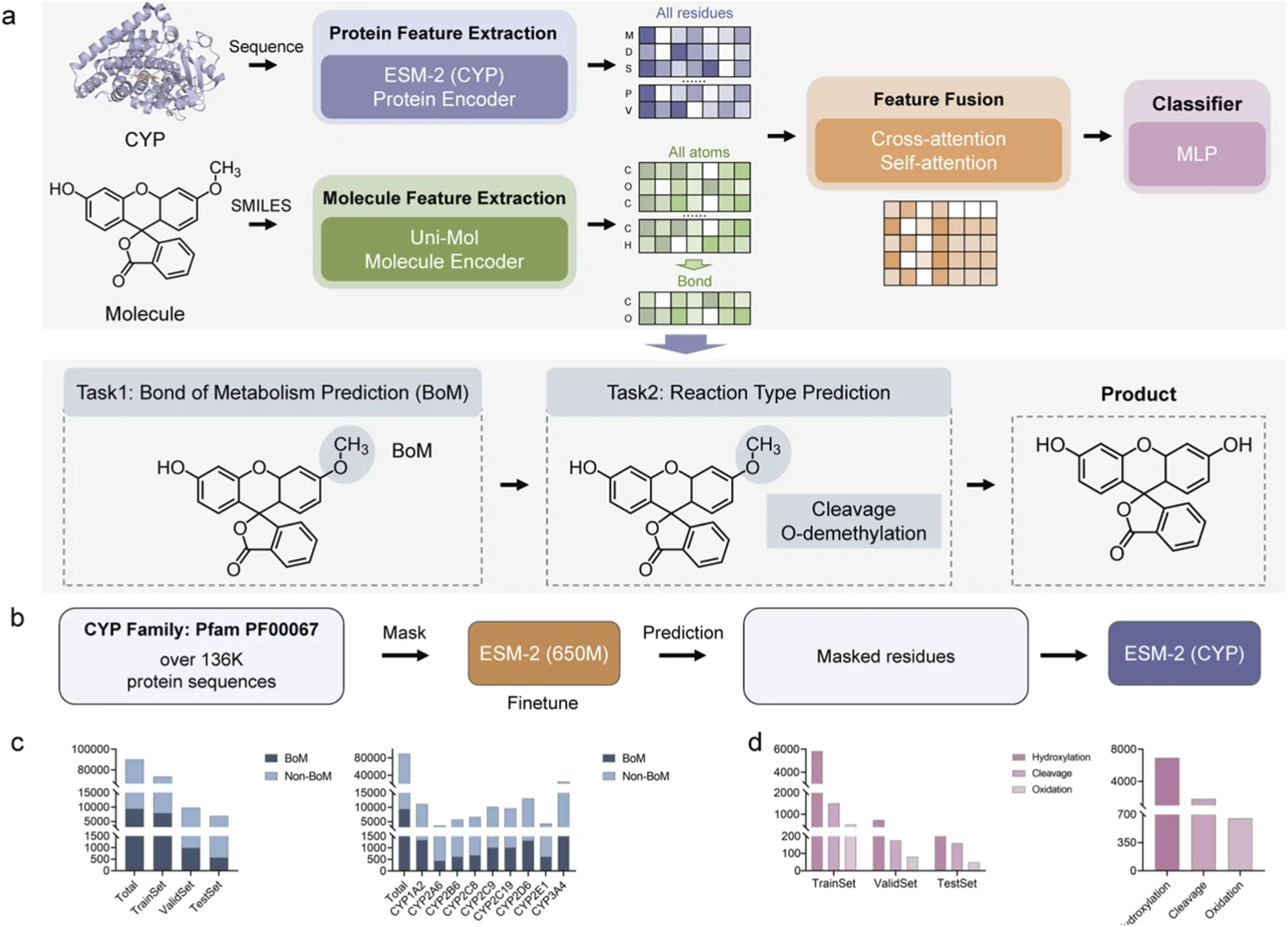
Overview of the MetaCYP framework and associated datasets. **(a)** Schematic of the MetaCYP framework. CYP sequences and substrate SMILES are embedded by ESM-2(CYP) and Uni-Mol, respectively, to produce full-length protein representations and bond-level molecular features. Features are fused via cross-attention and self-attention, then passed to an MLP for BoM and reaction type prediction. **(b)** ESM-2 fine-tuned on CYP family sequences via MLM to produce the CYP-specialized encoder ESM-2(CYP). **(c)** Positive/negative sample distribution in the BoM dataset (left) and per-CYP subset breakdown (right). **(d)** Reaction type sample distribution across train, validation, and test splits (left) and across the full ReactType dataset (right).

MetaCYP is a multimodal deep learning framework that integrates CYP protein sequences and small-molecule SMILES representations to predict two downstream tasks. The framework consists of three sequential components: a feature extraction module, a feature fusion module, and a classification head (Figure 1a).

#### 2.1.1 Feature extraction

Protein sequences and molecular SMILES were independently encoded using ESM-2 and Uni-Mol to obtain their respective representations (Lin et al., 2023; Zhou et al., 2023). ESM-2 is a large-scale protein language model (650 M parameters) pretrained on the UniRef50 database and can generate residue-level embeddings (Lin et al., 2023). Given that CYPs constituted only a small subset of the pretraining distribution, we further fine-tuned ESM-2 on CYP family sequences (PF00067) to obtain a domain-adapted model, termed ESM-2(CYP), which provided enhanced representations for CYP-specific sequence characteristics (Figure 1b). For small molecules, Uni-Mol was employed to generate atom-level embeddings (Zhou et al., 2023). Bond representations were constructed by concatenating the embeddings of the two atoms forming each chemical bond, enabling explicit encoding of local chemical structures.

#### 2.1.2 Feature fusion

To model protein–ligand interactions, multimodal representations were integrated via an attention-based fusion module comprising cross-attention and self-attention mechanisms (Vaswani et al., 2017). This design enabled the model to capture both inter-modal interactions and intra-representation dependencies in a unified framework. The attention mechanism computed dependencies between query (Q) and key (K) vectors, followed by SoftMax normalization to obtain attention weights, as Equation 1:

$$Attention\left(Q,K,V\right)=softmax\left(\;\frac{QK^{T}}{\sqrt{d_{k}}}\;\right)V$$ (1)

where Q, K, and V denote the query, key, and value matrices, respectively, and dk is the dimensionality of the key vectors used for scaling.

In the cross-attention layer, one modality served as the query while the other provided keys and values. Specifically, protein features attended to molecular features, enabling the model to identify biologically relevant interaction regions and capture binding-relevant correspondences between CYPs and substrates. The cross-attention operation is formulated as Equation 2:

$$Cross_{Attention\left(Q_{pro},K_{mol},V_{mol}\right)}=softmax\left(\;\frac{Q_{pro}{K_{mol}}^{T}}{\sqrt{d_{k}}}\;\right)V_{mol}$$ (2)

where: $Q_{pro}$ represent the query matrix from the protein features, $K_{mol}$ and $V_{mol}$ represent the key and value matrices from the small molecule features.

Following cross-modal interaction modeling, the resulting fused representation was further refined using self-attention. In this step, all queries, keys, and values were derived from the fused feature representation, allowing the model to capture global dependencies within the joint embedding space. The self-attention mechanism can be written as Equation 3:

$$Self_{Attention\left(Q_{fused},K_{fused},V_{fused}\right)}=softmax\left(\;\frac{Q_{fused}{K_{fused}}^{T}}{\sqrt{d_{k}}}\;\right)V_{fused}$$ (3)

F_fused_ denote the output of the cross-attention module, which integrates information from both protein and ligand modalities. Self-attention is then applied to F_fused_ to further refine contextual relationships and enhance representation expressiveness across the unified feature space.

#### 2.1.3 Downstream model

The fused protein–molecule representations were subsequently fed into a downstream classifier implemented as a multi-layer perceptron (MLP). The MLP consisted of two fully connected layers with a ReLU activation function applied between them. The final output layer produced class probabilities, yielding a 2-dimensional output for BoM prediction and a 3-dimensional output for reaction type classification.

For the BoM prediction task, we evaluated model performance using multiple metrics, including accuracy, balanced accuracy, precision, recall, F1 score, Jaccard score, MCC, ROC-AUC, and PR-AUC. These metrics provided a balanced assessment of classification performance from different perspectives, covering both threshold-dependent and ranking-based evaluations. For the ReactType prediction task, which was formulated as a three-class classification problem, we adopted macro-averaged metrics to ensure equal contribution from each class. Specifically, we computed macro-precision, macro-recall, macro-F1 score, and macro-Jaccard score, along with accuracy, balanced accuracy, MCC, ROC-AUC (one-vs-rest), and PR-AUC. The macro-averaging strategy mitigated the impact of class imbalance by averaging performance across classes independently. For PR-AUC computation, classes lacking either positive or negative samples were excluded to avoid undefined average precision values.

Although BoM prediction and reaction-type prediction were implemented using the same model architecture, the two tasks were trained independently on their respective datasets. Specifically, no shared trainable parameters were optimized across the two tasks, no joint loss function was used, and gradients from one task did not update the feature representations learned for the other task. During inference, the CYP–molecule pair was provided as input, and the separately trained BoM and reaction-type models were used to generate the corresponding predictions.

Accuracy quantifies the proportion of correctly classified samples among all predictions. It is defined based on the numbers of true positives (TP), true negatives (TN), false positives (FP), and false negatives (FN), and is given by Equation 4:

$$Accuracy=\frac{TP+TN}{TP+TN+FP+FN}$$ (4)

Balanced accuracy accounts for class imbalance by equally considering sensitivity and specificity. It is defined as the average of these two measures, providing a more reliable assessment than standard accuracy when class distributions are skewed, calculated according to Equation 5.

$$BalancedAccuracy=\frac{sensitivity+specificity}{2}$$ (5)

Precision measures the proportion of correctly predicted positive samples among all samples predicted as positive by the model, calculated according to Equation 6. It reflects the model’s ability to avoid false positives and is defined as the ratio of TP to the sum of TP and FP. Macro-precision is computed by first calculating precision for each class independently and then averaging across all classes.

$$Precision=\frac{TP}{TP+FP}$$ (6)

Recall quantifies the proportion of actual positive samples that are correctly identified by the model, calculated according to Equation 7. It reflects the model’s ability to capture positive instances and is defined as the ratio of TP to the sum of TP and FN. Macro-recall is defined as the average recall across all classes.

$$Recall=\frac{TP}{TP+FN}$$ (7)

F1 provides a harmonic mean of precision and recall, offering a single metric that balances both measures, calculated according to Equation 8. It is particularly useful when there is an uneven class distribution or when a trade-off between precision and recall needs to be considered. Macro-F1 score is obtained by computing the F1 score for each class separately and then averaging over all classes.

$$F1=2*\frac{Precision*Recall}{Precision+Recall}$$ (8)

Jaccard score quantifies the similarity between the predicted positive set and the ground-truth positive set, calculated according to Equation 9. It is defined as the ratio of the intersection to the union of the two sets, providing a strict measure of agreement between predictions and actual labels. Macro-Jaccard score is defined as the mean Jaccard index across all classes.

$$Jaccard=\frac{TP}{TP+FP+FN}$$ (9)

MCC evaluates the quality of classifications by considering all four outcomes: TP, TN, FP, and FN. It produces a balanced metric even in the presence of class imbalance, reflecting the correlation between predicted and actual labels, calculated according to Equation 10.

$$MCC=\frac{TP*TN‐FP*FN}{\sqrt{\left(TP+FP\right)\left(TP+FN\right)\left(TN+FP\right)\left(TN+FN\right)}}$$ (10)

ROC-AUC refers to the area under the Receiver Operating Characteristic (ROC) curve and is used to evaluate model performance across different decision thresholds. It summarizes the trade-off between the true positive rate and false positive rate, providing an overall measure of discriminative ability in binary classification.

PR-AUC denotes the area under the Precision–Recall (PR) curve and quantifies model performance over varying thresholds by jointly considering precision and recall. It is particularly informative for imbalanced datasets, as it focuses on the positive class and typically offers a more sensitive evaluation of minority-class detection compared to ROC-AUC.

### 2.2 Dataset

#### 2.2.1 BoM dataset

The dataset used in this study was derived from the dataset published in the GLMCyp study, which itself originated from Zaretzki’s dataset (Huang et al., 2025; Tian et al., 2021). The authors of GLMCyp further curated and refined the substrate reaction site dataset through multi-step pipeline. Specifically: (1) substrate molecules appearing in both the training and test sets were removed from the test set while all these substrates were retained in the training set; (2) annotations for identical reaction types were standardized across the dataset; and (3) the labeling rules were redefined to focus on the oxidation of key atoms such as carbon and nitrogen, rather than describing reactions solely based on alternating single and double bonds in structural formulas. After curation, the training, validation, and test sets contained 600, 76, and 71 substrate molecules, respectively, covering nine major CYPs, with a dataset split ratio of 8:1:1.

Furthermore, all chemical bonds within each substrate molecule were systematically enumerated to construct CYP–bond sample pairs. For each pair, a label of one was assigned if the bond in the substrate molecule was susceptible to CYP-catalyzed reactions, and 0 otherwise. As a result, the training, validation, and test sets contain 73,632, 9,802, and 6,890 CYP-bond samples, respectively. The distribution of positive and negative samples across the overall dataset and individual CYP-specific subsets is illustrated in Figure 1c.

#### 2.2.2 Reaction type dataset

The reaction type dataset was constructed by selecting samples labeled as one in the reaction site dataset across the training, validation, and test sets. These samples corresponded to chemical bonds within substrate molecules that was capable of being catalytically transformed by CYP enzymes (CYP–bond sample pairs). Each selected sample was then annotated with its corresponding reaction type, forming the training, validation, and test sets for reaction type prediction.

Each sample in this dataset likewise consisted of a chemical bond from a substrate molecule paired with one of nine human CYPs. The original reaction type annotations were manually reviewed and mapped into three coarse-grained categories according to the annotated reactive site and the major transformation pattern reported in the literature. Hydroxylation was retained as an independent class. Cleavage-related transformations included cleavage, desulfuration, dehydrogenation, denitrosation, and reduction. Oxidation-related transformations included oxidation, S-oxidation, N-oxidation, and epoxidation. This mapping was designed to reduce the severe label imbalance in the original annotations while preserving the major CYP-mediated transformation patterns. Future studies with larger and more balanced CYP metabolism datasets will be needed to support more detailed subtype-level reaction classification and metabolite-specific prediction. The numbers of samples in the training, validation, and test sets were 6,945, 1,842, and 653, respectively. The distribution of samples across reaction types is shown in Figure 1d.

## 3 Results and discussion

### 3.1 A unified model for BoM and reaction type prediction

Upon entering the CYP catalytic pocket, drug molecules interact with key active-site residues and the heme iron (Fe) center, enabling a range of oxidative transformations, including hydroxylation, dealkylation, and epoxidation (Hirao et al., 2025; Jiang et al., 2023). Both the functional residues lining the binding cavity and the structural features of the substrate jointly govern the binding pose, and consequently, the metabolic outcomen, encompassing both the BoM and the reaction type. Most existing SoM prediction models focus exclusively on substrate properties while ignoring enzyme identity, a design choice that forces separate models for each CYP isoform and prevents capture of enzyme-dependent metabolic variability (Tian et al., 2021). Building on our prior work, DeepP450 for CYP-specific substrate recognition and GLMCyp for BoM prediction (Chang et al., 2024; Huang et al., 2025), both of which achieved state-of-the-art performance on public benchmarks, we recognized that predicting metabolic sites alone is insufficient to fully characterize drug metabolism, since metabolite identity critically determines efficacy and toxicological risk.

Here we extend this framework into MetaCYP, a unified model that simultaneously predicts BoMs and reaction types across nine human CYPs, enabling downstream inference of metabolite structures (Figure 1a). CYP protein sequences and substrate SMILES serve as dual inputs; ESM-2 extracts global sequence-level enzyme representations (Lin et al., 2023), while Uni-Mol generates bond-level molecular features constructed by concatenating the representations of each bond’s two constituent atoms (Zhou et al., 2023). These heterogeneous features are then integrated through cross-attention and self-attention, and a MLP head predicts the BoM and reaction type. To improve the quality of protein representations, we fine-tuned ESM-2 (650 M) on CYP family sequences via masked language modeling (MLM), yielding a CYP-specialized encoder denoted ESM-2(CYP) (Figure 1b). Training data for BoM prediction were derived from the publicly available CyProduct dataset (Figure 1c) (Tian et al., 2021), and reaction type annotations within this dataset were further curated into three mechanistic categories, hydroxylation, bond cleavage, and oxidation, constituting the ReactType dataset (Figure 1d). Both datasets are class-imbalanced; full preprocessing procedures are described in the Methods.

MetaCYP achieved strong and consistent performance across both tasks (Table 1). On the BoMTest set, the model attained a balanced accuracy of 0.899, F1 of 0.761, MCC of 0.741, ROC-AUC of 0.956, and PR-AUC of 0.788, reflecting robust discrimination under class imbalance. Per-isoform performance was similarly stable, with MCC values spanning 0.618–0.877 and F1 scores spanning 0.643–0.887, confirming generalization across diverse enzyme–substrate metabolic contexts. On the reaction type task, MetaCYP achieved an overall MCC of 0.796 and ROC-AUC of 0.946, with per-isoform MCC of 0.675–0.865, F1 of 0.767–0.944, and ROC-AUC of 0.889–0.972. Particularly strong results were observed for the major drug-metabolizing isoforms CYP2C9, CYP2C19, CYP2D6, and CYP3A4, where BoM ROC-AUC ranged from 0.958 to 0.992 and reaction type ROC-AUC from 0.945 to 0.972, isoforms of foremost pharmacological relevance.

**TABLE 1:** MetaCYP performance on BoMTest (BoM prediction) and ReactTest (reaction type prediction).

Testset	acc	bacc	precision	recall	F1	MCC	jaccard	ROC-AUC	PR-AUC
BoMTest	0.957	0.899	0.704	0.829	0.761	0.741	0.615	0.956	0.788
ReactTest	0.895	0.818	0.933	0.818	0.862	0.796	0.761	0.946	0.922
Testset	1A2	2A6	2B6	2C8	2C9	2C19	2D6	2E1	3A4
MCC									
BoMTest	0.696	0.708	0.618	0.708	0.877	0.841	0.813	0.627	0.694
ReactTest	0.772	0.675	0.819	0.757	0.860	0.840	0.865	0.693	0.775
F1									
BoMTest	0.715	0.733	0.643	0.732	0.887	0.851	0.829	0.700	0.717
ReactTest	0.863	0.858	0.871	0.880	0.907	0.887	0.944	0.767	0.807
ROC-AUC									
BoMTest	0.921	0.925	0.958	0.963	0.970	0.992	0.958	0.925	0.961
ReactTest	0.938	0.889	0.964	0.907	0.962	0.970	0.972	0.972	0.945

We further performed an isoform-stratified evaluation on BoMTest and ReactTest set. MetaCYP achieved consistently high ROC-AUC values across all nine CYPs on BoMTest set, ranging from 0.921 to 0.992, indicating generally robust discriminative ability (Supplementary Table S1). However, performance varied across CYPs, with stronger results observed for CYP2C9 (MCC:0.877) and CYP2C19 (MCC:0.841) and relatively lower F1 scores and MCC values for CYP2B6 (F1:0.643; MCC:0.618) and CYP2E1 (F1:0.700; MCC:0.627). On ReactTest set, MetaCYP showed generally robust performance across the nine CYPs, with ROC-AUC values ranging from 0.889 to 0.972 and PR-AUC values ranging from 0.878 to 0.968 (Supplementary Table S2). Nevertheless, performance was not uniform across isoforms. CYP2D6 achieved the strongest overall performance (F1:0.944; MCC:0.865), whereas CYP2E1 showed relatively lower F1 (0.767) and recall (0.778). This variability may reflect differences in sample size, substrate diversity, and reaction-type composition among CYPs. To further evaluate the potential effect of reaction-type imbalance, we performed a reaction type-stratified analysis on ReactTest using a one-vs-rest strategy (Supplementary Table S3). MetaCYP achieved strong performance for hydroxylation, with an F1 score of 0.929 and PR-AUC of 0.966. Bond cleavage showed high precision but relatively lower recall (0.698), indicating that the model tended to make conservative predictions for this class and missed some true bond cleavage events. Oxidation also showed high overall discriminative ability, with an ROC-AUC of 0.980 and PR-AUC of 0.921, but its recall remained lower than that of hydroxylation (0.755). Although MetaCYP performs robustly across major reaction categories, reaction-type-dependent variability remains, likely reflecting differences in sample size and annotation density among reaction classes. Therefore, larger and more balanced datasets covering diverse CYPs and reaction pathways to further improve model robustness and generalizability.

**FIGURE 2:**
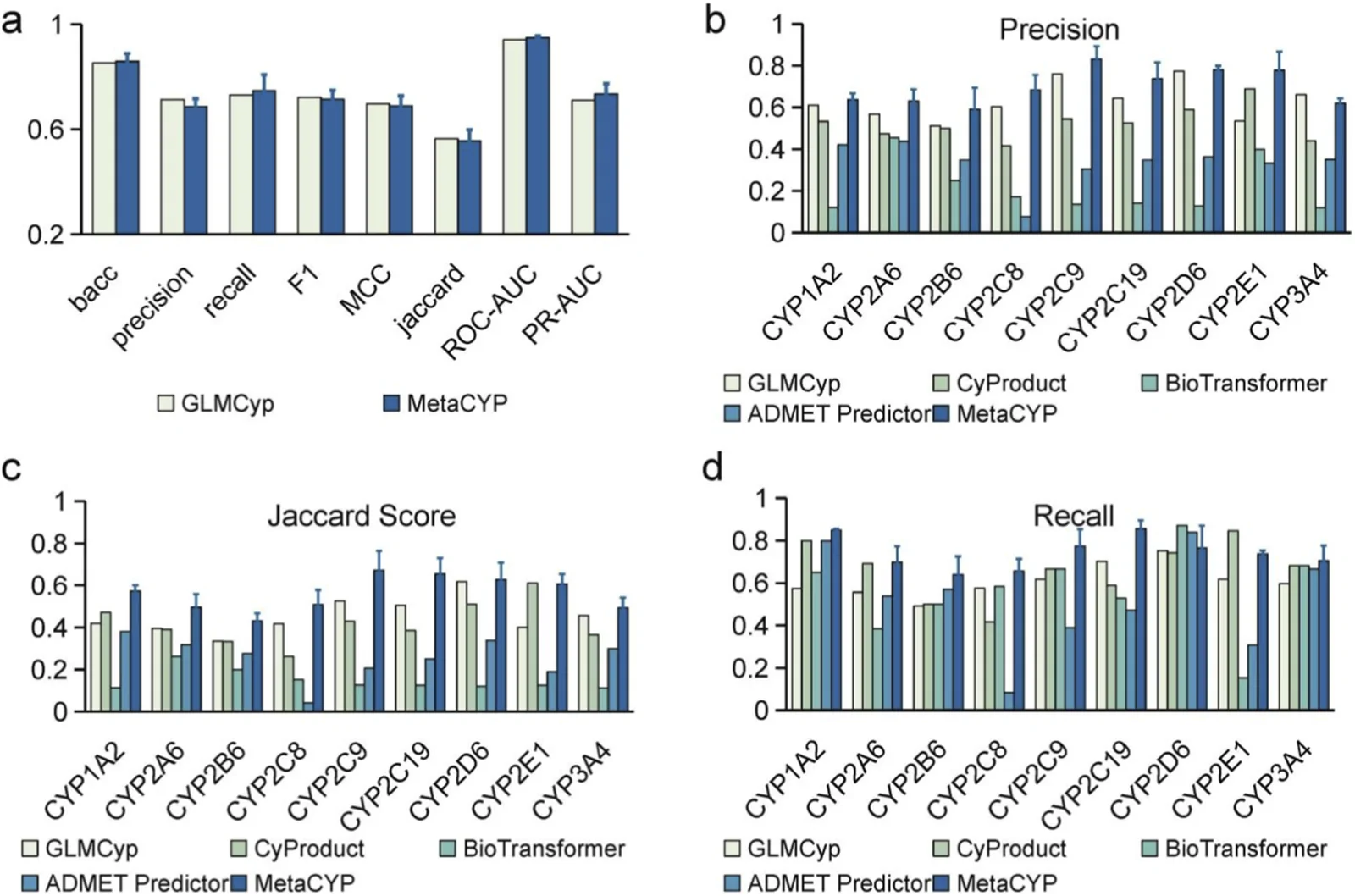
Comparative analysis on the BoMTest dataset. **(a)** Overall performance of MetaCYP and GLMCyp evaluated by balanced accuracy, precision, recall, F1, MCC, Jaccard score, ROC-AUC, and PR-AUC. Per-CYP comparison of precision **(b)**, Jaccard score **(c)**, and recall **(d)** across GLMCyp, CyProduct, BioTransformer, ADMET Predictor, and MetaCYP for nine human CYP subsets. (For MetaCYP, results are reported as mean ± standard deviation over three independent runs; error bars denote standard deviation).

To further evaluate model stability, we repeated all experiments three times using different random seeds and reported the mean ± standard deviation of each metric (Figure 2; Supplementary Tables S4–S6). Overall, MetaCYP exhibited low performance variability across repeated runs. For the BoM prediction task, the standard deviations of ROC-AUC ranged from 0.004 to 0.016, indicating highly reproducible performance. Similar trends were observed for reaction-type prediction, where most CYP isoforms showed stable results across different random initializations. Although slightly larger variations were observed for several low-sample-size isoforms, the consistently high ROC-AUC values suggest that the model maintains robust discriminative capability and captures stable CYP-specific metabolic patterns.

### 3.2 Benchmarking MetaCYP

Accurate BoM identification is a prerequisite for downstream reaction type annotation and underpins the reliability of the full CYP-mediated product prediction pipeline. We benchmarked MetaCYP against four widely used tools, GLMCyp, CyProduct, BioTransformer, and ADMET Predictor, on the BoMTest dataset (Figures 2a–d) (Huang et al., 2025; Simulations Plus, Inc, 2020; Tian et al., 2021; Wishart et al., 2022). While GLMCyp and BioTransformer achieved marginally higher precision (0.713 vs. 0.704) and recall (0.902 vs. 0.829), respectively, MetaCYP led on all remaining metrics: balanced accuracy 0.899, F1 0.761, MCC 0.741, and ROC-AUC 0.956 (Figures 2a,d, Supplementary Table S7). Relative to GLMCyp, the strongest baseline, MetaCYP improved consistently across F1, MCC, Jaccard score, and PR-AUC by margins of 0.040–0.099, reflecting sharper discrimination of true metabolic bonds and greater robustness under skewed class distributions. CyProduct tended to favor sensitivity over specificity, while BioTransformer and ADMET Predictor showed substantially weaker bond-level localization (Simulations Plus, Inc, 2020; Wishart et al., 2022).

At the isoform level, MetaCYP maintained competitive performance across all nine CYPs, recording the highest precision (0.581–0.887), Jaccard score (0.474–0.800), and recall (0.720–0.909) for most isoforms (Figures 2b–d; Supplementary Table S7). Improvements were most pronounced for CYP2C9, CYP2C19, CYP2D6, and CYP3A4, isoforms collectively responsible for the majority of clinically relevant xenobiotic metabolism, where MetaCYP exceeded GLMCyp by up to 0.156 in precision, 0.235 in Jaccard score, and 0.270 in recall across individual subsets. Together, these results establish MetaCYP as an accurate and generalizable BoM prediction framework, providing a robust foundation for downstream reaction type prediction and metabolite inference.

Representative cross-attention heatmaps showed that the model focuses on restricted and bond-dependent regions of the CYP sequence, rather than displaying diffuse attention across the entire protein. Notably, chemically equivalent C-H bonds exhibited nearly identical attention patterns and prediction scores, supporting the internal consistency of the model (Supplementary Figure S1).

### 3.3 Ablation study

To dissect the contribution of protein representation quality, we compared three encoder configurations within MetaCYP: the original pretrained ESM-2, the CYP-adapted ESM-CYP obtained by fine-tuning on CYP family sequences, and ESM-CYP-Finetune, in which ESM-CYP was further updated during downstream training (Table 2). On BoM prediction, ESM-CYP delivered the most balanced results: balanced accuracy 0.899, F1 0.761, MCC 0.741, Jaccard 0.615, PR-AUC 0.788, outperforming the base ESM-2 across most metrics. ESM-CYP-Finetune posted the highest raw accuracy (0.960) and ROC-AUC (0.960) but fell behind ESM-CYP in balanced accuracy (0.868) and recall (0.758), suggesting that continued end-to-end updating reinforces confidence on frequent bond patterns while eroding sensitivity to minority and structurally ambiguous metabolic sites. The same trend was sharper still on reaction type prediction, where ESM-CYP achieved an accuracy of 0.895, balanced accuracy of 0.818, F1 of 0.862, MCC of 0.796, and Jaccard of 0.761, decisively ahead of both alternatives. ESM-CYP-Finetune suffered the steepest decline, with balanced accuracy falling to 0.700 and MCC to 0.663, indicating substantially reduced generalization across the multi-class reaction space. These results collectively argue that CYP-adapted yet frozen representations strike a better balance than either generic encoders or aggressively task-optimized ones, validating MetaCYP’s design rationale of embedding enzyme-specific prior knowledge at the representation level while preserving the pretrained biochemical regularity needed for broad generalization.

**TABLE 2:** Model performance on BoMTest and ReactTest across ESM-2 encoder configurations: original pretrained ESM-2, CYP-adapted ESM-CYP, and ESM-CYP further updated during downstream training (ESM-CYP-Finetune).

MetaCYP performance on BoMTest									
Protein encoder	acc	bacc	precision	recall	F1	MCC	jaccard	ROC-AUC	PR-AUC
ESM-2	0.957	0.881	0.718	0.789	0.752	0.729	0.602	0.949	0.754
ESM-CYP	0.957	**0.899**	0.704	**0.829**	**0.761**	**0.741**	0.615	0.956	0.788
ESM-CYP-Finetune	**0.960**	0.868	**0.762**	0.758	0.760	0.738	0.613	0.960	0.790
MetaCYP performance on ReactTest									
ESM-2	0.885	0.768	**0.940**	0.768	0.828	0.773	0.713	0.971	0.930
ESM-CYP	**0.895**	**0.818**	0.933	**0.818**	**0.862**	0.796	0.761	0.946	0.922
ESM-CYP-Finetune	0.829	0.700	0.919	0.700	0.765	0.663	0.629	0.944	0.905

Bold values indicate the best performance for each metric.

Turning to feature integration, we replaced the attention-based fusion module with a simple concatenation of protein and bond-level molecular features and evaluated both tasks (Table 3). Attention-based fusion outperformed concatenation consistently. For BoM prediction, the substitution caused declines in balanced accuracy (−0.055), recall (−0.113), MCC (−0.061), and ROC-AUC (−0.026), demonstrating that flat feature stacking cannot resolve the fine-grained dependencies between enzyme sequence context and local bond chemistry that determine site selectivity. The penalty was far more severe for reaction type prediction: accuracy dropped from 0.895 to 0.697, precision from 0.933 to 0.512, F1 from 0.862 to 0.526, and MCC from 0.796 to 0.380. These figures confirm that the attention mechanism is not an architectural convenience but a functional necessity; it is what allows MetaCYP to learn the enzyme–substrate compatibility signals underlying both site identification and reaction assignment.

**TABLE 3:** Model performance on BoMTest and ReactTest comparing attention-based feature fusion against direct feature concatenation.

MetaCYP performance on BoMTest									
Feature fusion	acc	bacc	precision	recall	F1	MCC	jaccard	ROC-AUC	PR-AUC
Attention	**0.957**	**0.899**	**0.704**	**0.829**	**0.761**	**0.741**	**0.615**	**0.956**	**0.788**
Concatenation	0.951	0.844	0.698	0.716	0.707	0.680	0.547	0.930	0.726
MetaCYP performance on ReactTest									
Attention	**0.895**	**0.818**	**0.933**	**0.818**	**0.862**	**0.796**	**0.761**	**0.946**	**0.922**
Concatenation	0.697	0.564	0.512	0.565	0.526	0.380	0.435	0.699	0.624

Bold values indicate the best performance for each metric.

Finally, to quantify the value of enzyme information itself, we compared the full CYP–molecule model against a molecule-only variant that relies solely on bond-level substrate features (Table 4). For BoM prediction, excluding CYP representations degraded balanced accuracy (0.899 vs. 0.831), recall (0.829 vs. 0.690), MCC (0.741 vs. 0.666), and ROC-AUC (0.956 vs. 0.918), confirming that local bond reactivity, while informative, cannot account for isoform-specific site preference on its own. The disparity widened considerably for reaction type prediction, where the molecule-only model dropped across every metric: accuracy (0.895 vs. 0.763), balanced accuracy (0.818 vs. 0.602), F1 (0.862 vs. 0.650), MCC (0.796 vs. 0.498), Jaccard (0.761 vs. 0.499), ROC-AUC (0.946 vs. 0.812), and PR-AUC (0.922 vs. 0.728). Taken together, these ablations establish that reliable CYP metabolism prediction demands the explicit co-modeling of substrate reactivity and enzyme sequence-derived determinants, reinforcing the enzyme-aware design of MetaCYP over ligand-centric alternatives.

**TABLE 4:** Model performance on BoMTest and ReactTest for the full CYP-molecule model versus a molecule-only variant lacking CYP protein representations.

MetaCYP performance on BoMTest									
Feature representation	acc	bacc	precision	recall	F1	MCC	jaccard	ROC-AUC	PR-AUC
CYP-molecule	**0.957**	**0.899**	**0.704**	**0.829**	**0.761**	**0.741**	**0.615**	**0.956**	**0.788**
Molecule-only	0.949	0.831	0.697	0.690	0.694	0.666	0.531	0.918	0.678
MetaCYP performance on ReactTest									
CYP-molecule	**0.895**	**0.818**	**0.933**	**0.818**	**0.862**	**0.796**	**0.761**	**0.946**	**0.922**
Molecule-only	0.763	0.602	0.769	0.602	0.650	0.498	0.499	0.812	0.728

Bold values indicate the best performance for each metric.

### 3.4 Case study

**FIGURE 3:**
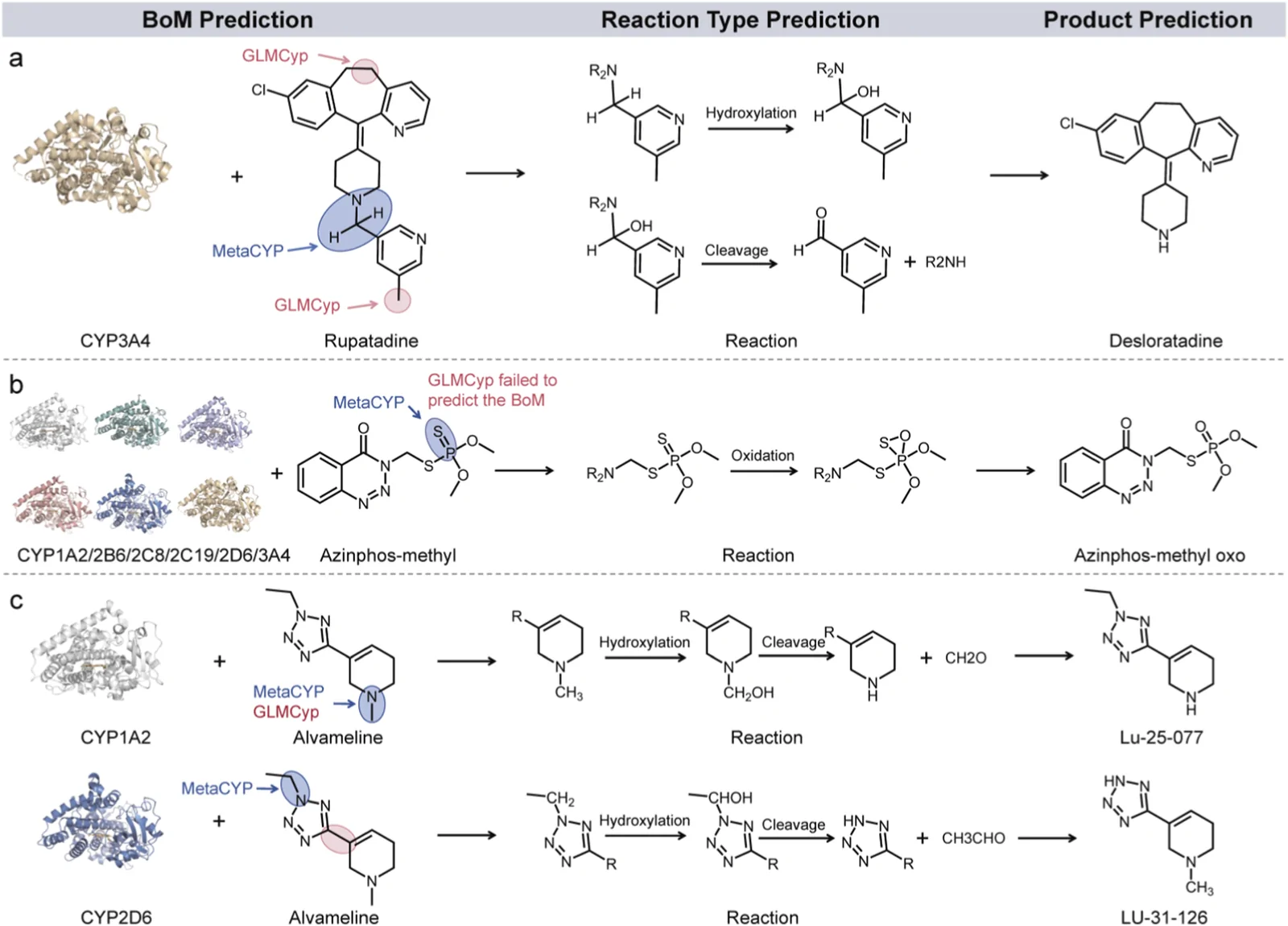
Case analysis of BoM and reaction type prediction. **(a)** For Rupatadine metabolized by CYP3A4, MetaCYP correctly assigns the BoM to the benzylic tertiary amine N-adjacent methylene (–CH_2_–N–) and predicts N-dealkylation via α-carbon hydroxylation and C–N bond cleavage, yielding Desloratadine. GLMCyp incorrectly places the BoM at an unreactive aromatic position. **(b)** For Azinphos-methyl across six isoforms (CYP1A2, 2B6, 2C8, 2C19, 2D6, and 3A4), MetaCYP correctly identifies the thioether sulfur (P=S) as the BoM and predicts S-oxidation (P=S → P=O), producing Azinphos-methyl oxon. GLMCyp detects no reactive site. **(c)** For Alvameline, MetaCYP resolves isoform-specific BoMs: under CYP1A2, the aliphatic tertiary amine α-carbon (–CH_2_–N–) undergoes hydroxylation and oxidative C–N bond cleavage to yield LU-25–077; under CYP2D6, the BoM shifts to the triazole-adjacent methylene, where hydroxylation and subsequent cleavage produce LU-31–126. GLMCyp recovers only the CYP1A2-associated BoM and fails to identify the CYP2D6-specific site.

To probe MetaCYP’s capacity to resolve enzyme-dependent regioselectivity and reaction mechanisms in practice, we examined three representative cases and compared predictions against GLMCyp (Huang et al., 2025). In the CYP3A4–Rupatadine system (Figure 3a), MetaCYP correctly identifies the benzylic N-adjacent methylene (–CH_2_–N–) as the primary BoM and predicts α-carbon hydroxylation followed by C–N bond cleavage, producing the known N-dealkylation product Desloratadine, a mechanistically sound assignment consistent with CYP3A4’s well-established preference for tertiary amine substrates. GLMCyp places the BoM at an aromatic position generally unreactive toward this transformation, reflecting an insufficient grasp of bond-level chemical reactivity.

For Azinphos-methyl (Figure 3b), MetaCYP correctly assigns the thioether sulfur (P=S) as the dominant BoM across six isoforms (CYP1A2, 2B6, 2C8, 2C19, 2D6, and 3A4) and predicts S-oxidation (P=S → P=O), in line with the established bioactivation of organophosphorus compounds to their oxon forms. GLMCyp detects no reactive site at all, exposing a fundamental blind spot for heteroatom-centered transformations beyond carbon. The most instructive example is Alvameline (Figure 3c), where MetaCYP resolves isoform-specific divergence: for CYP1A2, it identifies the aliphatic tertiary amine α-carbon and predicts hydroxylation-driven oxidative dealkylation, whereas for CYP2D6 it reassigns the BoM to the methylene linker adjacent to the triazole ring, capturing a distinct regioselective pattern rooted in enzyme–substrate complementarity. The two predictions yield structurally different metabolites, LU-25-077 and LU-31-126, respectively. GLMCyp recovers only the CYP1A2 site and misses the CYP2D6 locus entirely. Across all three cases, MetaCYP demonstrates not only superior BoM accuracy but also mechanistically coherent reasoning, encompassing heteroatom oxidation and isoform-level regioselectivity, capabilities that are indispensable for trustworthy metabolite prediction.

False-positive BoM recognition remains an important limitation of MetaCYP. Although the model achieved high recall in BoM prediction, its precision indicates that a fraction of predicted metabolic bonds were not experimentally annotated as true BoMs. Manual inspection of representative cases showed that some false positives occurred at bonds with local chemical environments similar to experimentally observed metabolic sites. For example, in CYP3A4-mediated rupatadine metabolism, MetaCYP assigned high probability to an additional bond located in a chemically similar environment to the true BoM (Supplementary Figure S2a). In carbosulfan, MetaCYP correctly identified the sulfur-associated site involved in S-oxidation by CYP3A4 but also predicted a five-membered-ring C–H bond as an additional BoM. Similar false-positive C–H predictions were also observed for CYP isoforms for which carbosulfan was not annotated as metabolized (Supplementary Figure S2b). These observations suggest that false-positive BoM predictions may arise from reactive or metabolism-like local substrate environments, as well as from limited modeling of CYP isoform-specific substrate recognition. Future integration of protein–ligand docking, structural constraints, metabolite annotations, and prospective experimental validation may help reduce false-positive BoM predictions and improve isoform-specific metabolic site identification.

### 3.5 Model generalizability

To further evaluate the predictive reliability of MetaCYP, we constructed two independent external validation datasets for the BoM prediction task (Zhang et al., 2024). The first validation set, BoM_CYP9, contained 3195 CYP–bond samples involving the same nine CYP isoforms used during model development. MetaCYP achieved an MCC of 0.611 and a ROC-AUC of 0.937 on this dataset, with a recall of 0.608 and a precision of 0.685 (Table 5). These results indicate that the model retained predictive ability on independent CYP–bond samples for the CYP isoforms covered during model development. We further evaluated the model on 3848 CYP–bond samples from other human CYP isoforms not included in the training dataset, BoM_NewCYP. On this new-CYP validation set, MetaCYP achieved a ROC-AUC of 0.897 and a recall of 0.527, suggesting that the model retained discriminative ability for unseen CYP isoforms, although with reduced sensitivity compared with the validation set involving the nine training CYPs (Table 5). This may reflect differences in sequence similarity, substrate coverage, and the limited availability of experimentally annotated BoM data.

**TABLE 5:** Performance of MetaCYP on external validset: BoM_CYP9, BoM_NewCYP and ReactType_CYP9.

External validset	acc	bacc	precision	recall	F1	MCC	jaccard	ROC-AUC	PR-AUC
BoM_CYP9	0.937	0.789	0.685	0.608	0.644	0.611	0.475	0.937	0.683
BoM_NewCYP	0.943	0.747	0.476	0.527	0.500	0.471	0.333	0.897	0.495
ReactType_CYP9	0.731	0.649	0.535	0.649	0.583	0.517	0.520	0.700	0.650

In addition to BoM prediction, we curated an external validation set for the prediction task based on BoM_CYP9, termed ReactType_CYP9. On this dataset, MetaCYP achieved a recall of 0.649 and a balanced accuracy of 0.731 (Table 5). These results provide additional support for the predictive reliability of MetaCYP, indicating that the model can not only identify CYP-mediated metabolic bonds in independent samples but also provide informative predictions of the corresponding reaction types. for less-studied CYPs.

## 3.6 Discussion

MetaCYP addresses a fundamental gap in CYP metabolism prediction: the failure of existing SoM models to incorporate enzyme identity. Conventional approaches ground their predictions in substrate chemical reactivity, molecular descriptors, or graph neural networks, while disregarding the active-site residues that govern isoform-specific catalytic selectivity, inherently limiting their cross-CYP generalization. MetaCYP overcomes this by pairing ESM-2-derived protein sequence representations with Uni-Mol bond-level molecular features, fusing both modalities through cross-attention to capture the enzyme–substrate interactions that dictate which bond is metabolized and how. This design enables a single model to resolve divergent metabolic outcomes for the same substrate across different CYP isoforms, a distinction that ligand-centric models structurally cannot make. MetaCYP equally sidesteps the coverage constraints of rule-based reaction type assignment: rather than mapping transformations through SMIRKS templates or predefined reaction rules, it learns the site-to-reaction-type relationship directly from data, yielding better scalability and generalization to novel scaffolds and rare metabolic pathways.

Three limitations warrant attention. First, MetaCYP operates on protein sequences and SMILES strings without explicit three-dimensional structural information, which may blunt its sensitivity to the geometric determinants of binding pose and site selectivity; incorporating 3D enzyme and substrate structures is a natural next step toward greater physical realism. Second, performance on rare reaction types and low-frequency CYP isoforms remains constrained by dataset size and annotation depth, a challenge amenable to transfer learning, few-shot strategies, or hybrid approaches that blend data-driven learning with targeted rule-based constraints. A further limitation is that MetaCYP does not directly model P450 isoform-dependent regioselectivity. Although the model predicts reactive bonds and broad reaction types for CYP–substrate pairs, it does not generate metabolite structures, distinguish regioisomeric products, or quantify isoform-specific product distributions. Future studies incorporating experimentally confirmed metabolite structures and isoform-resolved metabolic profiles will be needed to address this problem.

GLMCyp achieved a higher precision than MetaCYP (0.713 vs. 0.704), indicating that GLMCyp produced fewer false-positive BoM predictions among its predicted positives, whereas MetaCYP prioritized recovering more true metabolic bonds, as reflected by its higher recall (0.829 vs. 0.730). In early-stage drug discovery and metabolic liability assessment, avoiding false negatives is often particularly important because missing a true metabolic site may lead to overlooked metabolic instability or reactive metabolite risk. In contrast, false-positive metabolic sites can be further evaluated by downstream experimental validation or more detailed computational analyses. Therefore, the slightly lower precision of MetaCYP may represent a practically useful trade-off, as it enables the model to recover more true metabolic bonds while maintaining competitive precision and stronger overall performance.

Taken together, MetaCYP establishes a unified deep learning framework that models reaction sites and reaction types from enzyme–substrate information, offering a mechanistically grounded and interpretable tool for elucidating CYP catalytic selectivity. Its capacity to improve the accuracy of ADME property predictions, alongside its scalability across isoforms, positions it as a practical resource for early-stage drug screening, metabolic risk assessment, and rational drug design.

## 4 Conclusion

MetaCYP is a multimodal deep learning framework for the prediction of BoMs and reaction types across nine human CYPs. By integrating CYP protein sequence representations with bond-level substrate features through an attention-based fusion mechanism, the model captures isoform-specific catalytic selectivity within a single unified architecture, eliminating the need for separate per-isoform models and removing dependence on predefined reaction rules. MetaCYP outperforms existing methods on both prediction tasks, demonstrating superior generalization to novel chemical structures and complex metabolic contexts. Its scalability across multiple CYPs, combined with strong benchmark performance, makes it a compelling computational tool for early-stage drug discovery, ADME evaluation, and metabolic risk assessment.

## Data Availability

All relevant data supporting the key findings of this study are publicly available. All datasets utilized in this paper and source code can be found at https://github.com/CjmTH/MetaCYP.
