# Supplementary material for: MetaCYP: a unified framework for prediction of cytochrome P450 metabolic sites and reaction types via multimodal deep learning
Source: Front Chem. 2026 Aug 12;14:1869559. doi: 10.3389/fchem.2026.1869559 (PMC13506836; doi:10.3389/fchem.2026.1869559)
Supplement: Supplementary file 1 [file Supplementaryfile1.docx]

Supplementary Material

MetaCYP: A Unified Framework for Prediction of Cytochrome P450 Metabolic Sites and Reaction Types via Multimodal Deep Learning

Jiamin Chang^1^, Xuhai Huang^1^, Boxue Tian^1^*

^1^MOE Key Laboratory of Bioinformatics, State Key Laboratory of Molecular Oncology, Beijing Frontier Research Center for Biological Structure, School of Pharmaceutical Sciences, Tsinghua University, Beijing, 100084, China

**Table and Figure of Contents**

# Table S1. Performance Comparison of GLMCyp, Uni-Mol and MetaCYP on BoMTest; S2

# Table S2. Performance Comparison of MetaCYP and Uni-Mol on ReactTest; S3

# Table S3. Performance of MetaCYP on ReactTest stratified by reaction type; S4

# Table S4. Performance Comparison of MetaCYP on BoMTest and ReactTest; S5

# Table S5. Performance of MetaCYP on BoMTest; S6

# Table S6. Performance of MetaCYP on ReactTest; S7

# Table S7. Performance Comparison of GLMCyp, CyProduct, BioTransformer, ADMET Predictor and MetaCYP on BoMTest; S8

# Figure S1. Representative cross-attention heatmaps for positive CYP2D6-Desmethylcitalopram bond predictions; S9

# Figure S2. Case analysis of false positive BoM recognition. S10

Table S1. Performance Comparison of GLMCyp, Uni-Mol and MetaCYP on BoMTest

| Single CYP isozyme performance in BoMTest | | | | | | | | | |
| --- | --- | --- | --- | --- | --- | --- | --- | --- | --- |
|  | acc | bacc | precision | recall | F1 | MCC | jaccard | ROC-AUC | PR-AUC |
| GLMCyp | | | | | | | | | |
| 1A2 | 0.950 | 0.837 | **0.672** | 0.703 | 0.687 | 0.660 | 0.523 | **0.934** | 0.685 |
| 2A6 | 0.943 | 0.847 | 0.652 | 0.732 | 0.690 | 0.660 | 0.526 | **0.926** | 0.608 |
| 2B6 | 0.935 | 0.761 | 0.500 | 0.560 | 0.528 | 0.494 | 0.359 | 0.903 | 0.404 |
| 2C8 | 0.947 | 0.832 | 0.658 | 0.694 | 0.676 | 0.647 | 0.510 | 0.942 | 0.536 |
| 2C9 | 0.981 | 0.903 | **0.935** | 0.811 | 0.869 | 0.861 | 0.768 | 0.961 | **0.890** |
| 2C19 | 0.972 | 0.900 | 0.766 | 0.818 | 0.791 | 0.776 | 0.655 | 0.950 | 0.760 |
| 2D6 | 0.963 | 0.887 | 0.764 | 0.797 | 0.780 | 0.760 | 0.639 | **0.967** | 0.762 |
| 2E1 | 0.856 | 0.801 | 0.588 | 0.714 | 0.645 | 0.560 | 0.476 | 0.882 | 0.669 |
| 3A4 | **0.952** | 0.827 | **0.704** | 0.679 | 0.691 | 0.665 | 0.528 | 0.925 | 0.716 |
| Uni-Mol | | | | | | | | | |
| 1A2 | **0.952** | 0.896 | 0.662 | 0.828 | **0.736** | **0.715** | **0.582** | 0.911 | 0.623 |
| 2A6 | 0.917 | 0.592 | 0.615 | 0.195 | 0.296 | 0.315 | 0.174 | 0.902 | 0.532 |
| 2B6 | **0.946** | 0.730 | **0.632** | 0.480 | 0.546 | 0.523 | 0.375 | 0.949 | 0.615 |
| 2C8 | 0.935 | 0.750 | 0.633 | 0.528 | 0.576 | 0.543 | 0.404 | 0.852 | 0.543 |
| 2C9 | 0.952 | 0.827 | 0.706 | 0.679 | 0.692 | 0.666 | 0.529 | 0.934 | 0.714 |
| 2C19 | **0.978** | **0.957** | 0.788 | 0.932 | **0.854** | **0.846** | **0.745** | **0.992** | 0.894 |
| 2D6 | 0.957 | 0.900 | 0.715 | 0.831 | 0.769 | 0.624 | **0.748** | 0.962 | **0.831** |
| 2E1 | 0.886 | 0.779 | **0.739** | 0.607 | 0.667 | 0.603 | 0.500 | 0.841 | 0.759 |
| 3A4 | 0.948 | 0.826 | 0.687 | 0.679 | 0.683 | 0.655 | 0.519 | 0.953 | **0.768** |
| MetaCYP | | | | | | | | | |
|  | acc | bacc | precision | recall | F1 | MCC | jaccard | ROC-AUC | PR-AUC |
| 1A2 | 0.946 | **0.899** | 0.621 | **0.844** | 0.715 | 0.696 | 0.557 | 0.921 | **0.704** |
| 2A6 | **0.947** | **0.883** | **0.673** | **0.805** | **0.733** | **0.708** | **0.579** | 0.925 | **0.832** |
| 2B6 | **0.946** | **0.841** | 0.581 | **0.720** | **0.643** | **0.618** | **0.474** | **0.958** | **0.625** |
| 2C8 | **0.956** | **0.850** | **0.743** | **0.722** | **0.732** | **0.708** | **0.578** | **0.963** | **0.749** |
| 2C9 | **0.982** | **0.938** | 0.887 | **0.887** | **0.887** | **0.877** | **0.800** | **0.970** | 0.858 |
| 2C19 | **0.978** | 0.946 | **0.800** | **0.909** | 0.851 | 0.841 | 0.741 | **0.992** | **0.942** |
| 2D6 | **0.969** | **0.929** | **0.782** | **0.881** | **0.829** | **0.813** | 0.707 | 0.958 | 0.792 |
| 2E1 | **0.879** | **0.830** | 0.656 | **0.750** | **0.700** | **0.627** | **0.538** | **0.925** | **0.809** |
| 3A4 | 0.950 | **0.883** | 0.646 | **0.806** | **0.717** | **0.694** | **0.559** | **0.961** | 0.752 |

Table S2. Performance Comparison of MetaCYP and Uni-Mol on ReactTest

| Single CYP isozyme performance in ReactTest | | | | | | | | | |
| --- | --- | --- | --- | --- | --- | --- | --- | --- | --- |
|  | acc | bacc | precision | recall | F1 | MCC | jaccard | ROC-AUC | PR-AUC |
| MetaCYP | | | | | | | | | |
| 1A2 | **0.875** | **0.822** | **0.943** | **0.821** | **0.863** | **0.772** | **0.765** | **0.938** | **0.903** |
| 2A6 | **0.829** | **0.844** | **0.927** | **0.844** | **0.858** | **0.675** | **0.772** | **0.889** | **0.903** |
| 2B6 | **0.880** | **0.841** | **0.933** | **0.841** | **0.871** | **0.819** | **0.775** | **0.964** | **0.958** |
| 2C8 | **0.889** | **0.852** | **0.954** | **0.852** | **0.880** | **0.757** | **0.806** | **0.907** | **0.912** |
| 2C9 | **0.925** | **0.878** | **0.946** | **0.878** | **0.907** | **0.860** | **0.833** | **0.962** | **0.949** |
| 2C19 | **0.909** | **0.844** | **0.956** | **0.844** | **0.887** | **0.840** | **0.800** | **0.970** | **0.954** |
| 2D6 | **0.941** | **0.925** | **0.974** | **0.925** | **0.944** | **0.865** | **0.899** | **0.972** | **0.968** |
| 2E1 | **0.821** | **0.708** | **0.921** | **0.708** | **0.767** | **0.693** | **0.629** | **0.972** | **0.956** |
| 3A4 | **0.891** | **0.765** | **0.875** | **0.765** | **0.807** | **0.775** | **0.691** | **0.945** | **0.878** |
| Uni-Mol | | | | | | | | | |
| 1A2 | 0.781 | 0.611 | 0.743 | 0.611 | 0.648 | 0.567 | 0.505 | 0.788 | 0.714 |
| 2A6 | 0.610 | 0.333 | 0.203 | 0.333 | 0.253 | 0.000 | 0.203 | 0.324 | 0.363 |
| 2B6 | 0.600 | 0.544 | 0.536 | 0.544 | 0.539 | 0.360 | 0.406 | 0.816 | 0.727 |
| 2C8 | 0.583 | 0.375 | 0.360 | 0.375 | 0.362 | 0.142 | 0.263 | 0.739 | 0.600 |
| 2C9 | 0.811 | 0.678 | 0.921 | 0.678 | 0.742 | 0.655 | 0.598 | 0.766 | 0.717 |
| 2C19 | 0.773 | 0.628 | 0.859 | 0.628 | 0.680 | 0.586 | 0.529 | 0.809 | 0.738 |
| 2D6 | 0.839 | 0.750 | 0.874 | 0.750 | 0.799 | 0.607 | 0.679 | 0.888 | 0.865 |
| 2E1 | 0.571 | 0.333 | 0.190 | 0.333 | 0.242 | 0.000 | 0.190 | 0.661 | 0.607 |
| 3A4 | 0.818 | 0.632 | 0.861 | 0.632 | 0.686 | 0.605 | 0.547 | 0.850 | 0.788 |

Table S3. Performance of MetaCYP on ReactTest stratified by reaction type

|  | acc | bacc | precision | recall | F1 | MCC | jaccard | ROC-AUC | PR-AUC |
| --- | --- | --- | --- | --- | --- | --- | --- | --- | --- |
| Hydroxylation | 0.902 | 0.865 | 0.867 | **1.000** | **0.929** | 0.796 | **0.867** | 0.945 | **0.966** |
| Cleavage | 0.913 | 0.847 | **0.982** | 0.698 | 0.816 | 0.780 | 0.689 | 0.913 | 0.878 |
| Oxidation | **0.976** | **0.876** | 0.949 | 0.755 | 0.841 | **0.834** | 0.725 | **0.980** | 0.921 |

Table S4. Performance Comparison of MetaCYP on BoMTest and ReactTest (The results were evaluated over three independent experiments using different random seeds. Values are presented as mean ± standard deviation.)

|  | acc | bacc | precision | recall | F1 | MCC | jaccard | ROC-AUC | PR-AUC |
| --- | --- | --- | --- | --- | --- | --- | --- | --- | --- |
| BoMTest | 0.951±0.005 | 0.858±0.030 | 0.686±0.030 | 0.747±0.060 | 0.714±0.034 | 0.689±0.038 | 0.556±0.042 | 0.949±0.006 | 0.734±0.039 |
| ReactTest | 0.884±0.021 | 0.799±0.056 | 0.917±0.023 | 0.799±0.056 | 0.843±0.048 | 0.769±0.045 | 0.737±0.066 | 0.946±0.016 | 0.902±0.041 |

Table S5. Performance of MetaCYP on BoMTest (The results were evaluated over three independent experiments using different random seeds. Values are presented as mean ± standard deviation.)

| Single CYP isozyme performance in BoMTest | | | | | | | | | |
| --- | --- | --- | --- | --- | --- | --- | --- | --- | --- |
|  | acc | bacc | precision | recall | F1 | MCC | jaccard | ROC-AUC | PR-AUC |
| 1A2 | 0.949±0.005 | 0.903±0.006 | 0.638±0.029 | 0.849±0.007 | 0.728±0.022 | 0.709±0.022 | 0.573±0.027 | 0.926±0.004 | 0.708±0.064 |
| 2A6 | 0.936±0.011 | 0.829±0.038 | 0.630±0.056 | 0.699±0.075 | 0.661±0.055 | 0.628±0.061 | 0.496±0.062 | 0.930±0.006 | 0.735±0.069 |
| 2B6 | 0.943±0.011 | 0.802±0.036 | 0.592±0.102 | 0.640±0.086 | 0.602±0.034 | 0.579±0.037 | 0.431±0.036 | 0.944±0.012 | 0.590±0.106 |
| 2C8 | 0.946±0.010 | 0.815±0.032 | 0.684±0.072 | 0.657±0.057 | 0.670±0.063 | 0.641±0.069 | 0.508±0.070 | 0.968±0.010 | 0.753±0.048 |
| 2C9 | 0.969±0.010 | 0.880±0.041 | 0.832±0.062 | 0.774±0.080 | 0.800±0.065 | 0.785±0.070 | 0.672±0.092 | 0.968±0.008 | 0.799±0.042 |
| 2C19 | 0.968±0.010 | 0.916±0.021 | 0.737±0.078 | 0.856±0.039 | 0.790±0.055 | 0.777±0.057 | 0.656±0.074 | 0.983±0.009 | 0.855±0.073 |
| 2D6 | 0.962±0.008 | 0.873±0.052 | 0.780±0.019 | 0.766±0.105 | 0.770±0.061 | 0.750±0.064 | 0.629±0.079 | 0.953±0.016 | 0.770±0.065 |
| 2E1 | 0.908±0.021 | 0.843±0.010 | 0.778±0.090 | 0.738±0.016 | 0.754±0.038 | 0.701±0.052 | 0.607±0.048 | 0.938±0.012 | 0.810±0.052 |
| 3A4 | 0.940±0.006 | 0.833±0.036 | 0.620±0.024 | 0.705±0.072 | 0.659±0.042 | 0.628±0.048 | 0.493±0.048 | 0.948±0.011 | 0.709±0.031 |

Table S6. Performance of MetaCYP on ReactTest (The results were evaluated over three independent experiments using different random seeds. Values are presented as mean ± standard deviation.)

| Single CYP isozyme performance in ReactTest | | | | | | | | | |
| --- | --- | --- | --- | --- | --- | --- | --- | --- | --- |
|  | acc | bacc | precision | recall | F1 | MCC | jaccard | ROC-AUC | PR-AUC |
| 1A2 | 0.870±0.07 | 0.803±0.026 | 0.941±0.03 | 0.803±0026 | 0.850±0.019 | 0.761±0.015 | 0.744±0.029 | 0.958±0.016 | 0.921±0.023 |
| 2A6 | 0.813±0.041 | 0.732±0.175 | 0.792±0.197 | 0.732±0.175 | 0.742±0.180 | 0.626±0.103 | 0.654±0.188 | 0.931±0.032 | 0.935±0.025 |
| 2B6 | 0.853±0.100 | 0.819±0.113 | 0.908±0.067 | 0.819±0.113 | 0.841±0.109 | 0.774±0.156 | 0.743±0.158 | 0.951±0.023 | 0.933±0.038 |
| 2C8 | 0.889±0.00 | 0.809±0.061 | 0.902±0.074 | 0.809±0.061 | 0.836±0.062 | 0.754±0.003 | 0.748±0.082 | 0.961±0.040 | 0.931±0.050 |
| 2C9 | 0.899±0.018 | 0.837±0.032 | 0.939±0.010 | 0.837±0.032 | 0.876±0.023 | 0.815±0.031 | 0.785±0.036 | 0.951±0.013 | 0.919±0.023 |
| 2C19 | 0.917±0.028 | 0.854±0.052 | 0.959±0.013 | 0.854±0.052 | 0.893±0.041 | 0.853±0.050 | 0.814±0.064 | 0.972±0.009 | 0.945±0.026 |
| 2D6 | 0.952±0.008 | 0.939±0.010 | 0.979±0.003 | 0.939±0.010 | 0.955±0.008 | 0.890±0.018 | 0.918±0.013 | 0.980±0.006 | 0.976±0.007 |
| 2E1 | 0.750±0.127 | 0.653±0.191 | 0.724±0.255 | 0.653±0.191 | 0.668±0.214 | 0.527±0.274 | 0.558±0.217 | 0.925±0.036 | 0.904±0.046 |
| 3A4 | 0.873±0.018 | 0.742±0.046 | 0.867±0.031 | 0.742±0.046 | 0.783±0.046 | 0.734±0.042 | 0.663±0.052 | 0.912±0.047 | 0.835±0.062 |

Table S7. Performance Comparison of GLMCyp, CyProduct, BioTransformer, ADMET Predictor and MetaCYP on BoMTest

| Muti-metrics for BoMTest | | | | | | | | | |
| --- | --- | --- | --- | --- | --- | --- | --- | --- | --- |
|  | acc | bacc | precision | recall | F1 | MCC | jaccard | ROC-AUC | PR-AUC |
| GLMCyp | 0.955 | 0.852 | **0.713** | 0.730 | 0.721 | 0.697 | 0.564 | 0.941 | 0.710 |
| CyProduct | **--** | **--** | 0.707 | 0.755 | **--** | **--** | 0.575 | **--** | **--** |
| BioTransformer | **--** | **--** | 0.424 | **0.902** | **--** | **--** | 0.405 | **--** | **--** |
| ADMET Predictor | **--** | **--** | 0.443 | 0.614 | **--** | **--** | 0.346 | **--** | **--** |
| MetaCYP | **0.957** | **0.899** | 0.704 | 0.829 | **0.761** | **0.741** | **0.615** | **0.956** | **0.788** |
|  | 1A2 | 2A6 | 2B6 | 2C8 | 2C9 | 2C19 | 2D6 | 2E1 | 3A4 |
| jaccard | | | | | | | | | |
| GLMCyp | 0.420 | 0.395 | 0.335 | 0.418 | 0.525 | 0.506 | 0.618 | 0.401 | 0.457 |
| CyProduct | 0.471 | 0.391 | 0.333 | 0.263 | 0.429 | 0.385 | 0.511 | **0.611** | 0.366 |
| BioTransformer | 0.113 | 0.263 | 0.200 | 0.152 | 0.128 | 0.125 | 0.121 | 0.125 | 0.112 |
| ADMET Predictor | 0.381 | 0.318 | 0.276 | 0.042 | 0.206 | 0.250 | 0.338 | 0.190 | 0.299 |
| MetaCYP | **0.557** | **0.579** | **0.474** | **0.578** | **0.800** | **0.741** | **0.707** | 0.538 | **0.559** |
| precision | | | | | | | | | |
| GLMCyp | 0.610 | 0.568 | 0.512 | 0.604 | 0.760 | 0.644 | 0.773 | 0.535 | **0.661** |
| CyProduct | 0.533 | 0.474 | 0.500 | 0.417 | 0.545 | 0.526 | 0.590 | **0.688** | 0.441 |
| BioTransformer | 0.120 | 0.455 | 0.250 | 0.171 | 0.136 | 0.141 | 0.127 | 0.400 | 0.119 |
| ADMET Predictor | 0.421 | 0.438 | 0.348 | 0.077 | 0.304 | 0.348 | 0.364 | 0.333 | 0.351 |
| MetaCYP | **0.621** | **0.673** | **0.581** | **0.743** | **0.887** | **0.800** | **0.782** | 0.656 | 0.646 |
| recall | | | | | | | | | |
| GLMCyp | 0.574 | 0.556 | 0.491 | 0.576 | 0.619 | 0.702 | 0.752 | 0.619 | 0.597 |
| CyProduct | 0.800 | 0.692 | 0.500 | 0.417 | 0.667 | 0.588 | 0.742 | 0.846 | 0.683 |
| BioTransformer | 0.650 | 0.385 | 0.500 | 0.583 | 0.667 | 0.529 | 0.871 | 0.154 | 0.683 |
| ADMET Predictor | 0.800 | 0.538 | 0.571 | 0.083 | 0.389 | 0.471 | 0.839 | 0.308 | 0.667 |
| MetaCYP | **0.844** | **0.805** | **0.720** | **0.722** | **0.887** | **0.909** | **0.881** | **0.750** | **0.806** |

C-N bond as BoM

C-H bond as BoM

C-H bond as BoM

C-H bond as BoM

Figure S1. Representative cross-attention heatmaps for positive CYP2D6-Desmethylcitalopram bond predictions. Rows denote the bond-aware molecular query tokens (`mol_cls` and the two bond atoms), and columns denote CYP2D6 residues. Warmer colors indicate higher head-averaged cross-attention weights. The positive C-N bond candidate and three equivalent positive C-H bond candidates show concentrated, bond-dependent attention over limited sequence regions. The highly similar maps for the equivalent C-H bonds indicate stable and symmetry-consistent model behavior.

Figure S2. Case analysis of false positive BoM recognition.
